# New species of bone-eating worm *Osedax* from the abyssal South Atlantic Ocean (Annelida, Siboglinidae)

**DOI:** 10.3897/zookeys.814.28869

**Published:** 2019-01-08

**Authors:** Yoshihiro Fujiwara, Naoto Jimi, Paulo Y.G. Sumida, Masaru Kawato, Hiroshi Kitazato

**Affiliations:** 1 Department of Marine Biodiversity Research, Japan Agency for Marine-Earth Science and Technology, 2-15 Natsushima-cho, Yokosuka, Kanagawa 237-0061, Japan; 2 Department of Natural History Sciences, Graduate School of Science, Hokkaido University, N10 W8, Sapporo 060-0810, Japan; 3 Biological Oceanography Department, Oceanographic Institute – University of São Paulo, Praça do Oceanográfico, 191 - 05508-120, São Paulo-SP, Brazil; 4 Project Team for Analyses of Changes in East Japan Marine Ecosystems, Japan Agency for Marine-Earth Science and Technology, 2-15 Natsushima-cho, Yokosuka, Kanagawa 237-0061, Japan; 5 School of Marine Resources and Environment, Tokyo University of Marine Science and Technology, 4-5-7 Konan, Minato-ku, Tokyo 108-8477, Japan

**Keywords:** Polychaeta, São Paulo Ridge, taxonomy, whale-fall ecosystem

## Abstract

A new species of bone-eating annelid, *Osedaxbraziliensis***sp. n.**, found in a sunken whale carcass at a depth of 4,204 m at the base of the São Paulo Ridge in the South Atlantic Ocean off the Brazilian coast is described. The organism was retrieved using the human-occupied vehicle *Shinkai 6500* during the QUELLE 2013 expedition. This is the 26^th^ species of the genus and the first discovery from the South Atlantic Ocean, representing the deepest record of *Osedax* worldwide to date. This species morphologically resembles *Osedaxfrankpressi* but is distinguished by the presence of a yellow bump or patch behind the prostomium and its trunk length. Molecular phylogenetic analysis using three genetic markers (*COI*, *16S*, and *18S*) showed that *O.braziliensis***sp. n.** is distinct from all other *Osedax* worms reported and is a sister species of *O.frankpressi*.

## Introduction

Whale falls provide an extensive food supply to the oligotrophic deep-sea environments and harbour a unique biological assemblage, which is considered a “whale-fall ecosystem” (Smith et al. 1989, 2015). This ecosystem is known to be chemosynthesis-based, similar to hydrothermal vent and hydrocarbon seep ecosystems, but dynamic succession has been reported (Smith and Baco 2003). Numerous scavengers such as deep-sea sharks, hagfish, and small crustaceans devour the soft whale tissues when the carcasses reach the deep-sea floor (mobile-scavenger stage) (Smith and Baco 2003). After consumption of most soft tissues, organically enriched sediments and exposed bones are colonised by dense assemblages of opportunistic polychaetes and crustaceans (enrichment opportunist stage) (Smith and Baco 2003). Reducing chemicals such as sulphide are produced through anaerobic bacterial decomposition of organic materials in bones and support chemoautotrophs and chemoautotrophic symbiont-harbouring invertebrates as energy sources of primary production (sulphophilic stage) (Smith and Baco 2003). After the depletion of organic materials in the whale bones, the exhausted bones are thought to act as colonisation substrata primarily for suspension feeders (reef stage) but have never been observed in situ (Smith and Baco 2003).

One of the most unique organisms that has appeared in the whale-fall environment is an annelid polychaete of genus *Osedax* Rouse, Goffredi & Vrijenhoek, 2004 (Annelida, Siboglinidae), commonly known as bone-eating worms, discovered in whale carcasses in Monterey Bay (Rouse et al. 2004). Unlike other siboglinids, *Osedax* lack a discrete trophosome, the organ housing symbiotic bacteria in vestimentiferans and pogonophorans (Rouse et al. 2004). Instead, female *Osedax* possesses a vascularised “root” system penetrating the bone marrow (Rouse et al. 2004). *Osedax* worms are believed to acquire nutrition from the bones through their roots (Tresguerres et al. 2013, Miyamoto et al. 2017). Intracellular heterotrophic bacteria localise in the roots, but their role remains unclear (Goffredi et al. 2014). Surprisingly, all the visible worms are female, and the males are dwarfs, with the exception of one species (Rouse et al. 2015). Morphological characterisation of *Osedax* has been minimal thus far. The body size, palp colour, presence/absence of pinnules and their location on palps, presence/absence of oviduct and its length, and root form are examples of the limited characteristics used for species identification (Vrijenhoek et al. 2009).

To the best of our knowledge, 25 *Osedax* species have been described to date, and some additional undescribed species were reported from the West and East Pacific, North and South Atlantic, Mediterranean, Sub-Antarctic, and Antarctic (Rouse et al. 2004, Glover et al. 2005, Fujikura et al. 2006, Braby et al. 2007, Fujiwara et al. 2007, Goffredi et al. 2007, Rouse et al. 2008, Vrijenhoek et al. 2008, Vrijenhoek et al. 2009, Glover et al. 2013, Amon et al. 2014, Rouse et al. 2015, Taboada et al. 2015, Sumida et al. 2016, Rouse et al. 2018). Two species, i.e., *Osedaxrubiplumus* Rouse, Goffredi & Vrijenhoek, 2004 and *Osedaxfrankpressi* Rouse, Goffredi & Vrijenhoek, 2004, were first described from the bones of a grey whale carcass at a depth of 2,891 m in Monterey Bay, California, in 2004 (Rouse et al. 2004). *Osedaxmucofloris* Glover, Källström, Smith & Dahlgren, 2005 and *Osedaxjaponicus* Fujikura, Fujiwara & Kawato, 2006 were reported from environments with relatively shallow water at depths of 125 m in Swedish waters and 220 m near Japan, respectively (Glover et al. 2005, Fujikura et al. 2006). *Osedaxantarcticus* Glover, Wiklund, Taboada, Avila, Cristobo, Smith, Kemp, Jamieson & Dahlgren, 2013, *Osedaxcrouchi* Amon, Wiklund, Dahlgren, Copley, Smith, Jamieson & Glover, 2014, *Osedaxdeceptionensis* Taboada, Cristobo, Avila, Wiklund & Glover, 2013, *Osedaxnordenskjoeldi* Amon, Wiklund, Dahlgren, Copley, Smith, Jamieson & Glover, 2014, and *Osedaxrogersi* Amon, Wiklund, Dahlgren, Copley, Smith, Jamieson & Glover, 2014 were described from the Antarctic Ocean at depths between 10 and 1,446 m (Glover et al. 2013, Amon et al. 2014, Taboada et al. 2015). *Osedaxroseus* and *Osedaxpriapus* were reported from Monterey Bay, and the latter showed some unique morphological features, i.e., only two palps (four are typical) and no male dwarfism (Rouse et al. 2008, 2015). Recently, 14 species from Monterey Bay were simultaneously described (Rouse et al. 2018). Additionally, the genomes of several undescribed species were sequenced and deposited in the international nucleotide sequence databases.

In 2013, the Iatá-Piúna Expedition, a collaborative scientific cruise between Japan and Brazil, was conducted within the framework of the around-the-world research cruise Quelle 2013 (Quest for the Limit of Life) of Japan Agency for Marine-Earth Science and Technology (JAMSTEC) using the HOV *Shinkai 6500* (Sumida et al. 2016). A sunken whale carcass was discovered at a depth of 4,204 m at the base of the São Paulo Ridge in the South Atlantic Ocean (Sumida et al. 2016). This was the first record of a natural whale fall in the deep Atlantic Ocean (Sumida et al. 2016). Forty-one benthic taxa including many new species were documented from the carcass in which galatheid crabs, *Rubyspira* gastropods, and polychaete annelids were dominant (Silva et al. 2015, Sumida et al. 2016, Shimabukuro et al. 2017a, 2017b). The skeleton belonged to an Atlantic minke whale (*Balaenopterabonaerensis*) and was composed of nine caudal vertebrae, four of which were colonised by *Osedax* worms (Sumida et al. 2016, Alfaro-Lucas et al. 2017). Vertebrae not colonised by *Osedax* were well preserved and in a highly sulphophilic stage with chemosynthetic bacterial mats and numerous epifaunal organisms that fed on them. In contrast, vertebrae colonised by *Osedax* were heavily degraded and did not exhibit evidence of a sulphophilic stage, harbouring a distinct epifaunal assemblage (Alfaro-Lucas et al. 2017). A molecular phylogenetic analysis using mitochondrial *COI* sequences showed that the *Osedax* species from the São Paulo Ridge did not match any other sequences previously reported; therefore, the specimen was thought to be a new species.

Here we report a new species of *Osedax* collected from the South Atlantic at the deepest point recorded for this genus. Morphological and molecular phylogenetic characteristics are described.

## Materials and methods

### Specimen collection

**Figure 1.**
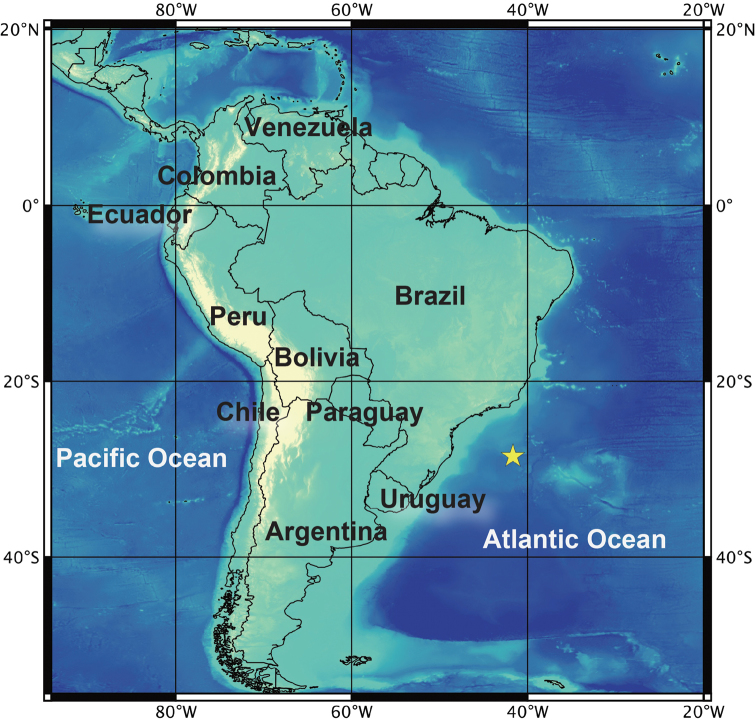
Sampling location of *Osedaxbraziliensis* sp. n. Solid star indicates the sampling location where a whale carcass was discovered at a depth of 4,204 m.

**Figure 2.**
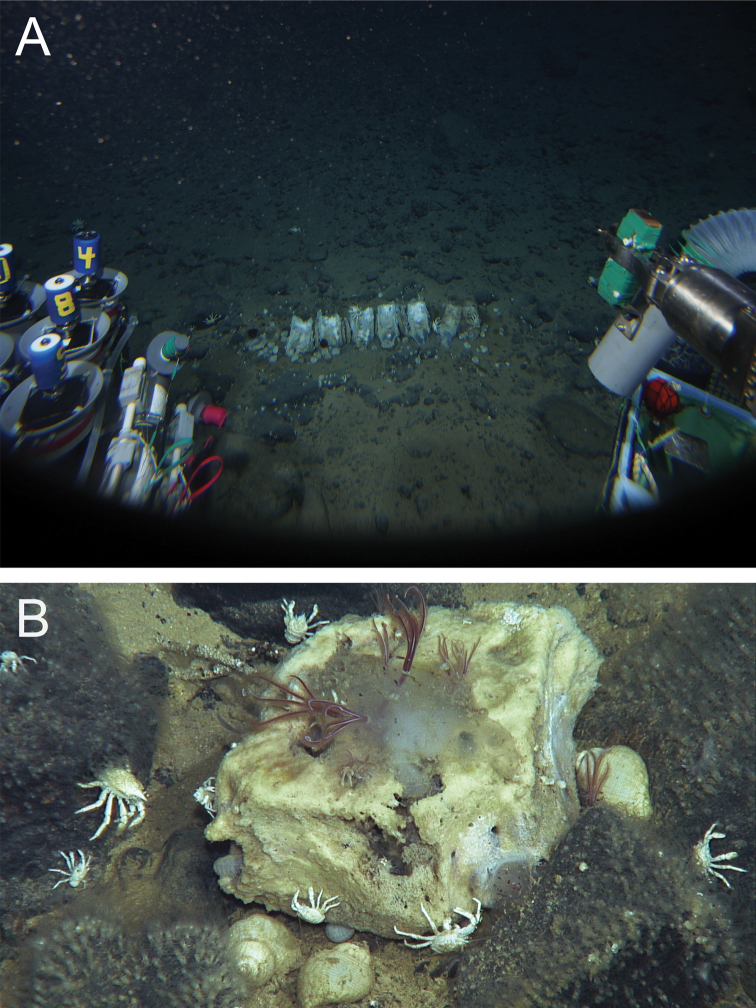
Whale skeleton discovered at a depth of 4,204 m in the South Atlantic Ocean. **A** Sunken whale skeleton of the Atlantic minke whale (*Balaenopterabonaerensis*). Seven vertebrae are visible in this field of view. *Osedaxbraziliensis* sp. n. had colonised the first two bones on the right **B** Close-up view of a vertebra colonized by *O.braziliensis* sp. n. Galatheid crabs (*Munidopsis* spp.), amphipods (*Stephonyx* sp.), and gastropods (*Rubyspira* sp.) were also seen on and around the bones.

Whale vertebrae harbouring *Osedax* worms were collected at a depth of 4,204 m at the base of the São Paulo Ridge (28°31.12'S, 41°39.41'W), southwest Atlantic Ocean during the HOV *Shinkai 6500* dives on April 24, 2013 (dive #1334), and April 26, 2013 (dive #1336), in the YK13-04 leg1 cruise using R/V *Yokosuka* (Figs 1–2). Upon recovery, the bones were immediately transferred to fresh chilled seawater (4 °C). *Osedax* worms were carefully removed from the bones under an on-board microscope just after the bone retrieval.

### Treatment for electron microscopic observation

Whole bodies of *Osedax* worms (*n* = 21) were fixed with 2.5% glutaraldehyde in filtered seawater for 24 h and preserved in filtered seawater with 10 mM sodium azide at 4 °C. Samples were then washed in filtered seawater and fixed with 2% osmium tetroxide in filtered seawater for 2 h at 4 °C. For scanning electron microscopic observation, each sample was rinsed with distilled water and incubated with 1% aqueous tannic acid (pH 6.8) for 1 h for conductive staining. These samples were again washed with distilled water and treated with 1% aqueous osmium tetroxide for 1 h. The worms were dehydrated in a graded ethanol series and critical point-dried using a JCPD-5 critical point dryer (JEOL, Akishima, Japan). The samples were coated with osmium using a POC-3 osmium plasma coater (MEIWAFOSIS Co., Osaka, Japan). The coated tissues were then observed using a JSM-6700F field-emission scanning electron microscope (JEOL) at an acceleration voltage of 5 kV.

### DNA preparation

DNA was extracted from the root tissues of nine the *Osedax* worms. To reduce surface contaminants, each tissue sample was thoroughly washed in autoclaved and filtered (0.22 µm) seawater. DNA extraction from tissue samples was conducted separately using the DNeasy Tissue Kit (Qiagen Japan, Tokyo, Japan), following the instruction provided by the manufacturer.

### Polymerase chain reaction (*PCR*) amplification, cloning, and sequencing

PCR amplifications were conducted using an Ex Taq PCR kit (TaKaRa, Kyoto, Japan) for three different molecular markers: cytochrome *c* oxidase subunit I (*COI*), 16S rRNA (*16S*), and *18S* rRNA (*18S*). Two oligonucleotide primers (0.2 µM each) and <1 µg of DNA template were added to the reaction mixtures. Thermal cycling was performed as follows: denaturing at 96 °C for 20 s, annealing at 55 °C for 45 s, and extension at 72 °C for 2 min for a total of 35 cycles. The oligonucleotide primer sequences used for the PCR amplification are shown in Table 1. The molecular sizes of the PCR products were confirmed with 1.2% Agarose S (Nippon Gene, Toyama, Japan) gel electrophoresis. The PCR products were purified using the Wizard SV Gel and PCR Clean-Up System (Promega, Madison, WI, USA). The DNA sequencing reaction was performed using a BigDye Terminator v3.1 Cycle Sequencing Kit (Applied Biosystems, Foster City, CA, USA). Specific primers for each gene were used in sequencing reactions according to the manufacturer’s recommended procedure (Table 1). Sequencing was performed using an ABI PRISM 3100 genetic analyser (Applied Biosystems).

**Table 1.** List of primers used for PCR and sequencing.

Primer	Target gene	Sequence (5'–3')	Application	Reference
P_CO1f	*COI*	TCMACTAATCAYAARGAYATTGGNAC	PCR, Sequencing	Nelson and Fisher (2000)
P_CO1r	*COI*	CCDCCTAGWCCTARRAARTGTTGNGG	PCR, Sequencing	Nelson and Fisher (2000)
Euk_42F	18S rRNA	CTCAARGAYTAAGCCATGCA	PCR, Sequencing	Takishita et al. (2007)
Euk_1520R	18S rRNA	CYGCAGGTTCACCTAC	PCR, Sequencing	Takishita et al. (2007)
Euk_555F	18S rRNA	AGTCTGGTGCCAGCAGCCGC	Sequencing (internal)	Takishita et al. (2007)
Euk_555R	18S rRNA	GCGGCTGCTGGCACCAGACT	Sequencing (internal) (Complement of Euk_555F)	Modified from Takishita et al. (2007)
Euk_1269R	18S rRNA	AAGAACGGCCATGCACCAC	Sequencing (internal)	Takishita et al. (2007)
16SarL	16S rRNA	CGCCTGTTTAACAAAAACAT	PCR, Sequencing	Palumbi et al. (2000)
16SbrH	16S rRNA	CCGGTCTGAACTCAGATCACGT	PCR, Sequencing	Palumbi et al. (2000)

### Phylogenetic analysis

Partial sequences of the *COI*, *16S*, and *18S* genes were analysed using the gapped-BLAST search algorithm (Altschul et al. 1997, Benson et al. 2000) to estimate the degree of similarity to other related sequences. Additional sequences of siboglinids for phylogenetic analyses were obtained from the non-redundant nucleotide sequence database of the DNA Data Bank of Japan (Kodama et al. 2018) (Table 2). Sequences were aligned using CLUSTAL X (Larkin et al. 2007), followed by automatic editing of the resulting alignments using the GBLOCKS program for all the genetic markers under the options allowing smaller final blocks, gap positions within the final blocks, and less strict flanking positions (Castresana 2000, Talavera and Castresana 2007). The alignments (34 OTUs / 3,112 bp in total) are available upon request from the corresponding author. The maximum likelihood (ML) analysis was performed using the RAxML-VI-HPC program (Stamatakis 2006). Evolutionary models for each marker (GTR + γ) were separately estimated using KAKUSAN4 software (Tanabe 2007). The ML bootstrap analyses (1,000 replicates, -f option) were constructed as in the model and using the settings described earlier in this section.

**Table 2.** List of operational taxonomic units included in the phylogenetic analysis, together with accession numbers in DDBJ.

Taxon	*COI*	16S rRNA	18S rRNA
* Lamellibrachia columna *	DQ996645	FJ347646	FJ347679
* Riftia pachyptila *	KJ789166	KP119573	KP119591
* Sclerolinum brattstromi *	KJ789167	FJ347645	FJ347680
* Osedax antarcticus *	KF444422	KF444418	KF444420
*Osedaxbraziliensis* sp. n.	LC381421	LC381422	LC381424
* Osedax bryani *	JX280610	KP119580	KP119593
* Osedax crouchi *	KJ598038	KJ598032	KJ598035
* Osedax deceptionensis *	KT860545	KF444419	KF444421
* Osedax docricketts *	FJ347626	FJ347650	FJ347688
* Osedax frankpressi *	FJ347607	FJ347658	FJ347682
* Osedax jabba *	FJ347638	FJ347647	FJ347693
* Osedax japonicus *	FM998111	LC381423	FM995535
* Osedax knutei *	FJ347635	FJ347648	FJ347692
* Osedax lehmani *	EU223323	FJ347660	FJ347689
* Osedax lonnyi *	FJ347643	FJ347651	FJ347695
* Osedax mucofloris *	KJ806976	N.A.	AY941263
* Osedax nordenskjoeldi *	KJ598033	KJ598033	KJ598036
* Osedax packardorum *	FJ347629	FJ347661	FJ347690
* Osedax priapus *	KP119564	KP119575	KP119594
* Osedax randyi *	FJ347615	FJ347659	FJ347684
* Osedax rogersi *	KJ598040	KJ598034	KJ598037
* Osedax roseus *	FJ347609	FJ347657	FJ347683
* Osedax rubiplumus *	EU852488	FJ347656	FJ347681
* Osedax ryderi *	KP119563	KP119574	KP119597
* Osedax sigridae *	FJ347642	FJ347655	FJ347694
* Osedax talkovici *	FJ347621	FJ347654	FJ347685
* Osedax tiburon *	FJ347624	FJ347653	FJ347687
* Osedax ventana *	EU236218	FJ347652	FJ347686
* Osedax westernflyer *	FM998110	FJ347649	FJ347691
*Osedax* sp. 'MB16'	JX280612	KP119581	KP119592
*Osedax* sp. 'mediterranea'	KT860548	KT860551	KT860550
*Osedax* sp. 'sagami3'	FM998081	N.A.	FM995537
*Osedax* sp. 'sagami4'	FM998082	N.A.	FM995541
*Osedax* sp. 'sagami5'	FM998083	N.A.	FM995539

### *COI* genetic distance

Minimum genetic distances based on Kimura 2 parameters (K2P) model were calculated between *Osedax* species using MEGA7 software (Kumar et al. 2016). These distances were calculated using the *COI* alignment used in the phylogenetic analyses without gaps.

## Systematics

### Family Siboglinidae Caullery, 1914

#### Genus *Osedax* Rouse, Goffredi & Vrijenhoek, 2004

##### Osedax braziliensis sp. n.

Taxon classificationAnimaliaSabellidaSiboglinidae

http://zoobank.org/7AD6CE45-4585-42C6-BB5E-22587BB2307F

**Figure 3.**
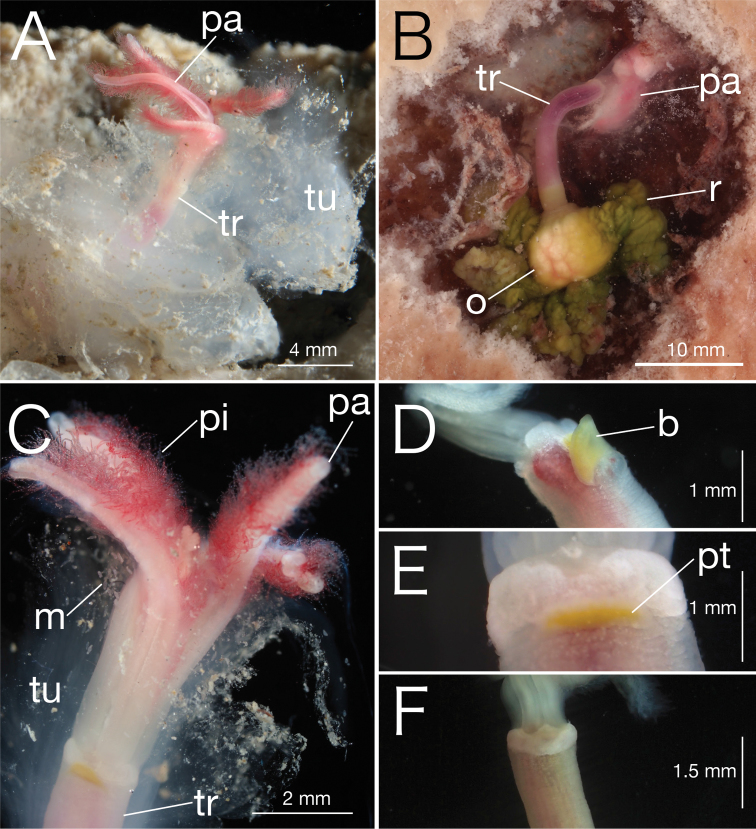
Photographs of unfixed *Osedaxbraziliensis* sp. n. **A** Palps *(pa)*, trunk *(tr)* and a gelatinous hemispherical tube *(tu)***B** lateral view of palps *(pa)*, trunk *(tr)*, ovary *(o)*, and root *(r)***C** ventral view of palps *(pa)* with pinnules *(pi)* and trunk *(tr).* Dwarf males *(m)* inhabit a gelatinous tube *(tu)*, and **D** ventro-lateral view of an individual possessing a yellow bump *(b)* present behind prostomium **E** Ventral view of holotype (NMST-Pol H-685) possessing a yellow patch *(pt)***F** Ventral view of an individual without yellow bump.

**Figure 4.**
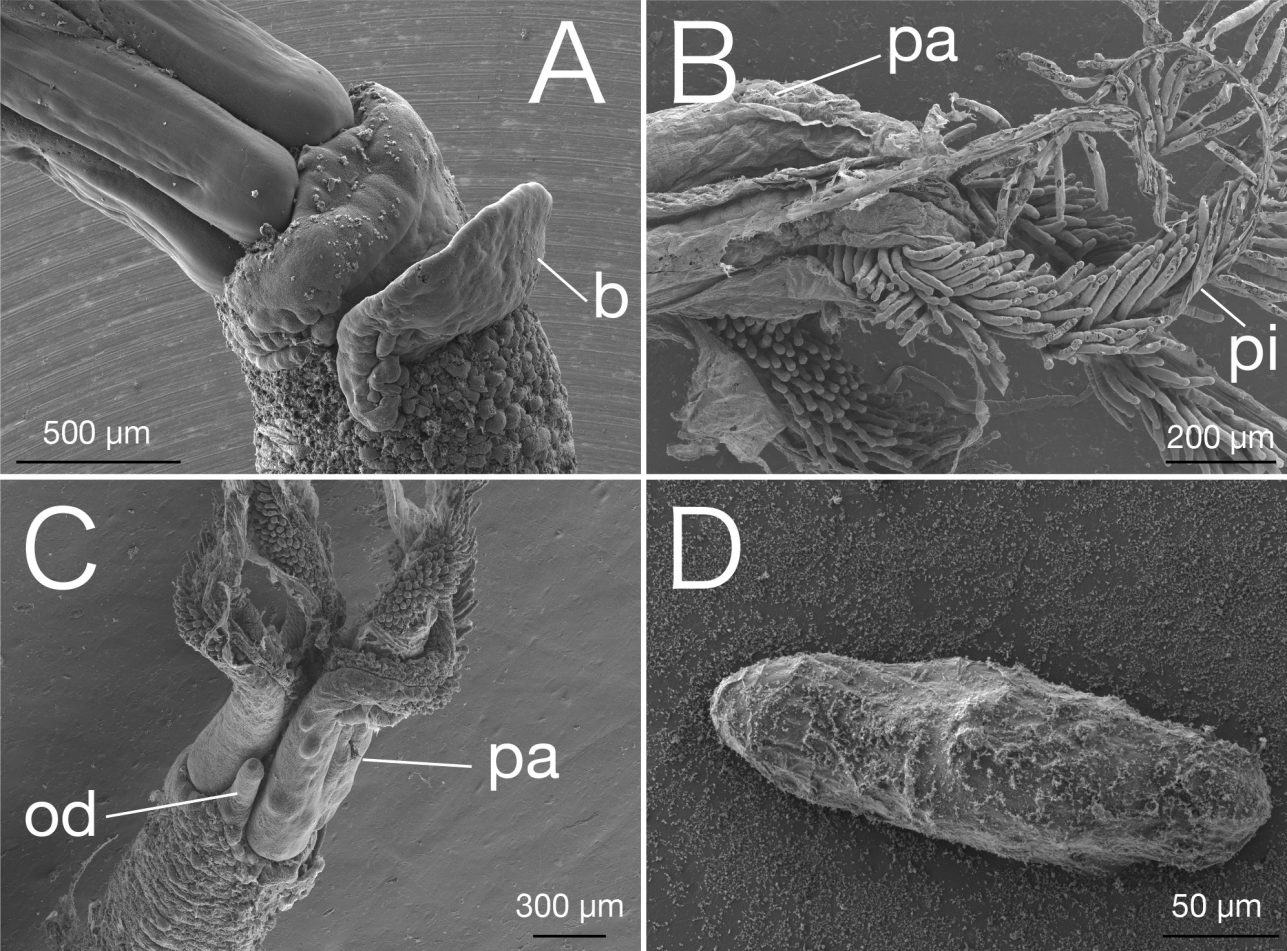
Scanning electron micrographs of *Osedaxbraziliensis* sp. n. **A** Ventro-lateral view of bump *(b)***B** lateral view of palps *(pa)* with pinnules *(pi)*, and **C** dorsal view of palps *(pa)* and oviduct *(od)***D** Dwarf male taken from the tube of a female.

Figures 3 , 4 New Japanese name: Burajiru-honekuihanamushi

Osedax sp. nov.: Sumida et al. 2016: 1–6, figs 3–4, Table 1. Osedax: Alfaro-Lucas et al. 2017: 1–9, fig. 2B.

###### Type material.

Holotype: NMST-Pol H-685, trunk 14 mm long, 2 mm wide, female, 4,203 m depth, 26 April 2013, collected by YF, DDBJ No. LC381421, LC381422, LC381424. Paratypes (14 specimens): NSMT-Pol P-686–690 and JAMSTEC-1130038806, 1130039105, 1130039113, 1130039116, 1130039146, 1130039163, 11 specimens, female, 24 and 26 April 2013, collected by YF, DDBJ No. LC381777, LC381766, LC381767, LC381768, LC381769, LC381771; JAMSTEC-1130057454, 1130057457, 1130057458, 3 specimens, male, 26 April 2013, collected by YF.

###### Type locality.

São Paulo Ridge, Brazil, 4,204 m depth.

###### Diagnosis.

Trunk length long. Gelatinous hemispherical tube encases trunk and base of palps. Yellow bump or patch present (absent in some specimens). Pinnules on inner margin of palps. Root lobulated without branching.

###### Description.

Genetic data (*COI*, *16S*, and *18S*) deposited in DDBJ (LC106303, LC381421, LC381422, LC381424, and LC381765–LC381787). Trunk length up to 22 mm, width at collar 0.5 mm, reddish purple while alive and whitish after fixation (Fig. 3A–C); gelatinous hemispherical tube encases trunk and base of palps, 1–2 mm thick, contains eggs and dwarf males (Fig. 3A, C). Prostomium whitish while alive, present at top of trunk. Yellow bump or patch present behind prostomium: this yellow bump or patch size varies among individuals, biggest bump reaches top of trunk, and is absent in some specimens (Fig. 3D–F). Crown consists of four palps; palps about 1.5 mm length, red colour while alive with two whitish stripes on the inner side, fused for about 30% of length; pinnules on inner margin of palps, about 50–250 µm, 7–8 pinnules in transverse rows (Figs 3A, C, 4B, C). Oviduct free to base, adjoined to the trunk at opposite side of prostomium region, reaching up to 20–30% of palp length (Fig. 4C). Ovisac whitish; trunk–ovisac junction about 15% of trunk length, light green while alive (Fig. 3B). Root lobulated without branching, yellow greenish while alive and whitish after fixation; intracellular symbiotic bacteria in root tissue (Fig. 3B). Eggs about 150 µm diameter (*n* = 20), whitish while alive (Fig. 3B).

Dwarf male about 250 µm in length (*n* = 20), fusiform, whitish while alive, prostomium and pygidium rounded, no appendage organs; posterior hooks present, two pairs (5–7 hooks per bundle) arranged in three rows (Figs 3C, 4D).

###### Etymology.

This species is named after the type locality, Brazil. This name is an adjective used as a substantive in the genitive case.

###### Distribution.

Only known from a whale carcass of the type locality. São Paulo Ridge, off Brazilian coast, 4,204 m depth.

###### Phylogenetic analysis.

**Figure 5.**
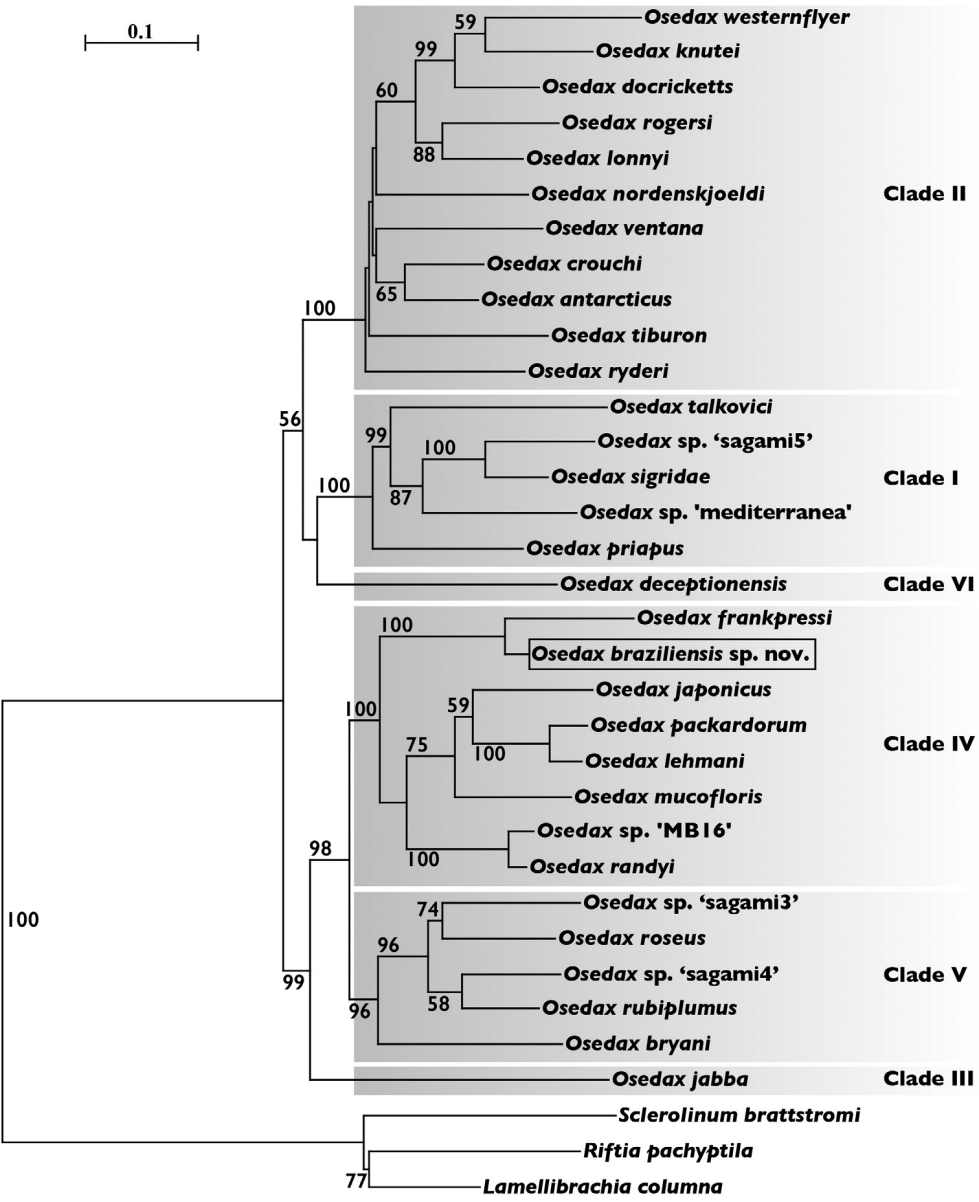
Phylogenetic placement of *Osedaxbraziliensis* sp. n. based on nucleotide sequences on the concatenated *COI*, *16S*, and *18S* markers, using maximum likelihood. Scale bar represents 0.1 nucleotide substitutions per sequence position. Only bootstrap values greater than 50 are shown for each branch. *Osedaxbraziliensis* sp. n. is boxed.

The final lengths of the aligned sequences were 1,004 bp (*COI*), 486 bp (*16S*), and 1,604 bp (*18S*). The phylogenetic position of *O.braziliensis* sp. n. determined from our ML analysis recovered, with total support, a distinct species from that of all other *Osedax* species reported (Fig. 5). The six *Osedax* clades proposed by Rouse et al. (2018) were recovered. The phylogenetic analysis showed that *O.braziliensis* sp. n. falls into Clade IV, and is a sister species of *O.frankpressi* known from Monterey Bay at depths between 1,820 m and 2,898 m (Fig. 5).

###### Remarks.

This species resembles *Osedaxfrankpressi* in the pinnules distributed only at the inner margin of palps, lobulated root structure without branching, gelatinous hemispherical tube, and dwarf males (Rouse et al. 2004). However, it can be discriminated from *O.frankpressi* by the presence of the yellow bump or patch behind the prostomium, trunk length, and genetic data. In *O.braziliensis* sp. n., the yellow bump or patch was present in some specimens including holotype, and the trunk length is long (6–22 mm), whereas in *O.frankpressi*, the bump or patch is absent in all specimens, and the trunk length is shorter (4.5 mm). *COI* genetic distances between *O.braziliensis* sp. n. and *O.frankpressi* are 0.111–0.117, which are greater than intraspecific values in *O.braziliensis* sp. n. (0.001–0.006). Genetic distances between *O.braziliensis* and the rest of the *Osedax* taxa for the *COI* ranged from 0.117 to 0.236 (Table 3).

**Table 3.** COI divergence values (Kimura 2 parameters) between *Osedax* species and OTUs.

	OTU	1	2	3	4	5	6	7	8	9	10	11	12	13	14	15	16	17	18	19	20	21	22	23	24	25	26	27	28	29	30	31	32	33	34
1	* Osedax frankpressi *																																		
2	*Osedax* sp. ‘sagami8’	0.230																																	
3	* Osedax tiburon *	0.206	0.188																																
4	*Osedax* sp. ‘sagami7’	0.207	0.221	0.241																															
5	* Osedax knutei *	0.238	0.167	0.231	0.230																														
6	*Osedax* sp. ‘sagami4’	0.193	0.214	0.214	0.162	0.212																													
7	* Osedax mucofloris *	0.198	0.224	0.209	0.204	0.190	0.214																												
8	* Osedax antarcticus *	0.209	0.209	0.218	0.235	0.211	0.201	0.255																											
9	* Osedax talkovici *	0.225	0.219	0.234	0.244	0.214	0.220	0.220	0.267																										
10	* Osedax randyi *	0.207	0.219	0.244	0.006	0.233	0.165	0.209	0.231	0.238																									
11	* Osedax crouchi *	0.209	0.213	0.180	0.178	0.196	0.177	0.203	0.205	0.221	0.180																								
12	*Osedax* sp. ‘MB16’	0.207	0.230	0.253	0.057	0.219	0.178	0.212	0.240	0.236	0.059	0.175																							
13	* Osedax sigridae *	0.192	0.198	0.190	0.214	0.207	0.207	0.230	0.212	0.215	0.208	0.188	0.217																						
14	* Osedax lehmani *	0.193	0.229	0.211	0.178	0.223	0.175	0.178	0.264	0.202	0.175	0.200	0.175	0.195																					
15	* Osedax ventana *	0.193	0.187	0.165	0.217	0.196	0.208	0.225	0.221	0.220	0.222	0.155	0.222	0.207	0.228																				
16	* Osedax roseus *	0.172	0.209	0.201	0.209	0.230	0.168	0.206	0.196	0.226	0.201	0.222	0.211	0.201	0.191	0.203																			
17	* Osedax lonnyi *	0.230	0.187	0.236	0.206	0.204	0.185	0.227	0.168	0.233	0.208	0.188	0.200	0.209	0.222	0.201	0.195																		
18	*Osedaxbraziliensis* sp. n.	0.117	0.203	0.203	0.184	0.211	0.217	0.196	0.193	0.236	0.183	0.206	0.190	0.187	0.217	0.198	0.170	0.214																	
19	* Osedax rubiplumus *	0.190	0.211	0.222	0.195	0.230	0.161	0.230	0.206	0.224	0.203	0.183	0.203	0.203	0.190	0.228	0.185	0.193	0.211																
20	* Osedax rogersi *	0.230	0.187	0.236	0.206	0.204	0.185	0.227	0.168	0.233	0.208	0.188	0.200	0.209	0.222	0.201	0.195	0.000	0.214	0.193															
21	*Osedax* sp. ‘mediterranea’	0.214	0.241	0.212	0.206	0.264	0.217	0.241	0.259	0.203	0.201	0.227	0.190	0.155	0.206	0.230	0.192	0.238	0.199	0.241	0.238														
22	* Osedax packardorum *	0.201	0.216	0.208	0.185	0.237	0.170	0.181	0.258	0.191	0.177	0.219	0.199	0.201	0.082	0.219	0.196	0.227	0.219	0.196	0.227	0.196													
23	* Osedax bryani *	0.209	0.213	0.180	0.178	0.196	0.177	0.203	0.205	0.221	0.180	0.000	0.175	0.188	0.200	0.155	0.222	0.188	0.206	0.183	0.188	0.227	0.219												
24	* Osedax nordenskjoeldi *	0.203	0.190	0.002	0.244	0.230	0.217	0.209	0.218	0.237	0.246	0.177	0.255	0.188	0.214	0.163	0.204	0.238	0.201	0.220	0.238	0.214	0.211	0.177											
25	* Osedax westernflyer *	0.233	0.004	0.188	0.221	0.170	0.211	0.224	0.212	0.222	0.219	0.216	0.230	0.200	0.229	0.193	0.206	0.193	0.205	0.211	0.193	0.244	0.216	0.216	0.190										
26	* Osedax ryderi *	0.209	0.213	0.180	0.178	0.196	0.177	0.203	0.205	0.221	0.180	0.000	0.175	0.188	0.200	0.155	0.222	0.188	0.206	0.183	0.188	0.227	0.219	0.000	0.177	0.216									
27	*Osedax* sp. ‘sagami6’	0.193	0.175	0.185	0.238	0.152	0.219	0.203	0.203	0.225	0.233	0.175	0.224	0.221	0.236	0.153	0.222	0.178	0.198	0.220	0.178	0.233	0.224	0.175	0.183	0.177	0.175								
28	* Osedax jabba *	0.228	0.172	0.222	0.203	0.206	0.251	0.195	0.238	0.228	0.209	0.216	0.228	0.222	0.241	0.188	0.220	0.213	0.208	0.243	0.213	0.227	0.227	0.216	0.219	0.172	0.216	0.206							
29	* Osedax docricketts *	0.198	0.172	0.183	0.236	0.157	0.219	0.206	0.206	0.225	0.230	0.170	0.227	0.218	0.233	0.148	0.222	0.176	0.201	0.220	0.176	0.236	0.222	0.170	0.180	0.175	0.170	0.008	0.203						
30	* Osedax priapus *	0.217	0.191	0.186	0.206	0.210	0.180	0.230	0.211	0.231	0.209	0.160	0.195	0.155	0.195	0.183	0.198	0.178	0.211	0.219	0.178	0.175	0.227	0.160	0.184	0.196	0.160	0.191	0.198	0.188					
31	*Osedax* sp. ‘sagami5’	0.193	0.214	0.207	0.222	0.204	0.206	0.212	0.228	0.226	0.222	0.198	0.224	0.122	0.203	0.209	0.217	0.219	0.203	0.198	0.219	0.183	0.214	0.198	0.204	0.217	0.198	0.212	0.214	0.207	0.180				
32	*Osedax* sp. ‘sagami3’	0.196	0.206	0.242	0.209	0.219	0.181	0.217	0.220	0.186	0.201	0.206	0.209	0.211	0.194	0.234	0.187	0.224	0.199	0.176	0.224	0.214	0.202	0.206	0.242	0.209	0.206	0.229	0.238	0.229	0.241	0.225			
33	* Osedax japonicus *	0.201	0.238	0.220	0.175	0.236	0.172	0.173	0.216	0.204	0.172	0.188	0.165	0.206	0.149	0.206	0.185	0.211	0.188	0.203	0.211	0.206	0.141	0.188	0.223	0.238	0.188	0.213	0.213	0.211	0.201	0.200	0.212		
34	* Osedax deceptionensis *	0.214	0.230	0.236	0.273	0.235	0.271	0.246	0.234	0.221	0.273	0.242	0.270	0.209	0.233	0.236	0.241	0.236	0.211	0.250	0.236	0.239	0.233	0.242	0.236	0.236	0.242	0.243	0.261	0.240	0.240	0.248	0.251	0.230	

## Supplementary Material

XML Treatment for Osedax braziliensis
